# Interplay of DDP4 and IP-10 as a Potential Mechanism for Cell Recruitment to Tuberculosis Lesions

**DOI:** 10.3389/fimmu.2018.01456

**Published:** 2018-07-05

**Authors:** Thomas Blauenfeldt, Linda Petrone, Franca del Nonno, Andrea Baiocchini, Laura Falasca, Teresa Chiacchio, Vincent Bondet, Valentina Vanini, Fabrizio Palmieri, Gianni Galluccio, Armanda Casrouge, Jesper Eugen-Olsen, Matthew L. Albert, Delia Goletti, Darragh Duffy, Morten Ruhwald

**Affiliations:** ^1^Center for Vaccine Research, Statens Serum Institut, Copenhagen, Denmark; ^2^Translational Research Unit, Department of Epidemiology and Preclinical Research, “L. Spallanzani” National Institute for Infectious Diseases (INMI), Rome, Italy; ^3^Pathology Unit, “L. Spallanzani” National Institute for Infectious Diseases (INMI), Rome, Italy; ^4^Laboratory of Electron Microscopy, Department of Epidemiology and Preclinical Research, “L. Spallanzani” National Institute for Infectious Diseases (INMI), Rome, Italy; ^5^Institut Pasteur, Laboratoire Immunobiologie des Cellules Dendritiques, Département d’Immunologie, Paris, France; ^6^INSERM U1223, Institut Pasteur, Paris, France; ^7^Clinical Department, “L. Spallanzani” National Institute for Infectious Diseases (INMI), Rome, Italy; ^8^Pneumology, Ospedale San Camillo, Rome, Italy; ^9^Copenhagen University Hospitals, Clinical Research Centre, Hvidovre, Denmark; ^10^Genentech Inc, South San Francisco, CA, United States

**Keywords:** tuberculosis, pulmonary, CXCL10, DPP4/CD26, bronchoalveolar lavage fluid, cytokines

## Abstract

**Introduction:**

*Mycobacterium tuberculosis* is one of the world’s most successful pathogens equipped to establish itself within the human host as a subclinical infection without overt disease. Unable to eradicate the bacteria, the immune system contains the infection in a granuloma structure. Th1 cells that are essential for infection control are recruited to the site of infection directed by chemokines, predominantly CXCL10. It has previously been shown that CXCL10 in the plasma of patients chronically infected with hepatitis C virus is present primarily in an antagonist form. This is due to N-terminal truncation by the enzyme DPP4, which results in the antagonist form that is capable of binding its receptor CXCR3, but does not induce signaling. We aimed to explore whether such CXCL10 antagonism may have an impact on the pathogenesis of tuberculosis (TB).

**Results:**

We measured plasma levels of agonist and antagonist CXCL10 by Simoa digital ELISA, as well as DPP4 enzyme activity in the plasma of 20 patients with active TB infection, 10 patients with pneumonia infection, and a group of 10 healthy controls. We found higher levels of total and antagonist CXCL10 and reduced DPP4 enzyme activity in the plasma of TB patients compared to controls. We traced the source of CXCL10 secretion using immunohistochemical and confocal analysis to multinucleated giant cells in the TB lesions, and variable expression by macrophages. Interestingly, these cells were associated with DPP4-positive T cells. Moreover, the analysis of lymphocytes at the site of TB infection (bronchoalveolar lavage) showed a reduced frequency of CXCR3^+^ T cells.

**Interpretation:**

Our data suggests that CXCL10 antagonism may be an important regulatory mechanism occurring at the site of TB pathology. CXCL10 can be inactivated shortly after secretion by membrane bound DPP4 (CD26), therefore, reducing its chemotactic potential. Given the importance of Th1 cell functions and IFN-γ-mediated effects in TB, our data suggest a possible unappreciated regulatory role of DPP4 in TB.

**Perspectives:**

DPP4 is the target for a class of enzyme inhibitors used in the treatment of diabetes, and the results from this study suggest that these drugs could be repurposed as an adjunct immunotherapy of patients with TB and MDR-TB.

## Introduction

*Mycobacterium tuberculosis* has co-evolved with humans for millennia (1). This has resulted in one of the world’s most successful bacterial pathogens equipped to establish itself within the human host for decades as a subclinical infection without overt disease. Today almost two billion people worldwide are latently infected with *M. tuberculosis* and 10.4 million people are estimated to have had acute pulmonary tuberculosis disease (TB) in 2015 of which 1.8 million people died (2).

Based on our current understanding, the immune system is unable to eradicate most *M. tuberculosis* infections, and a successful outcome is a protective immune response containing the infection in the granuloma structure. The immune response necessary for *M. tuberculosis* containment is complex and incompletely understood. Effective T cell responses are essential to eliminate bacteria and contain the infection; however, *M. tuberculosis* seems also to benefit from recognition by T cells possibly by driving immune exhaustion (3–5). Regulatory immune mechanisms are, therefore, important to balance control of inflammation and prevent pathology (6–8). Clonal expansion of *M. tuberculosis*-specific T effector cells occurs in adjacent lymph nodes, from where antigen-specific Th1 cells migrate to the lung in a chemokine-dependent manner (9). These cells produce IFN-γ leading to macrophage activation, cytokine and chemokine production, induction of microbicidal factors and bacterial control (10). CXCL10 (IP-10) released by infected alveolar macrophages is considered an important chemokine responsible for the directed migration of these Th1 cells to the site of infection (6, 11–13).

In line with its cardinal role in immune control of *M. tuberculosis* infection, CXCL10 has emerged as a potential correlate for treatment efficacy as well as a measure of TB disease severity and correlate of risk (14–19). Similarly, CXCL10 plasma levels are elevated in many other Th1-type inflammatory diseases (20) such as chronic hepatitis C viral (HCV) infection in which CXCL10 is an IL-28B independent predictive marker for the failure to respond to antiviral treatment (21). Casrouge and colleagues demonstrated that the high levels of CXCL10 found in patients chronically infected with HCV is due to the presence of predominantly an antagonist form, following amino-terminal truncation by the dipeptidyl dipeptidase 4 enzyme (DPP4) (22). In more recent work, we have shown therapeutic inhibition of DPP4 results in increased levels of agonist CXCL10 in the plasma of both healthy controls and chronic HCV patients (23). In addition, an experimental mouse model revealed that inhibition of DPP4 lead to reduced tumor burden by favoring the intra-tumor migration of effector Th1 cells (24).

DPP4 is a pleotropic protease best known for its central role in glucose metabolism responsible for the degradation of incretins such as GLP-1 (25). In the immune system, the membrane-bound form of DPP4, CD26, has co-stimulatory functions through direct interaction with ADA and CD45, as well as regulatory functions by inactivating proinflammatory mediators including chemokines and cytokines (25, 26). DPP4 truncation of CXCL10 generates a dominant negative form of the protein, which is capable of binding its receptor CXCR3 (CD183) but does not induce signaling (22, 23, 27).

To date, there has been no evidence to support a role for CXCL10 antagonism in mycobacterial disease pathogenesis despite numerous studies supporting its potential as a biomarker for TB disease (14, 16, 18, 28). Therefore, the aim of this study was to determine if CXCL10 antagonism is a feature of active TB, and moreover to establish evidence for whether CXCL10 deactivation is a potential regulatory mechanism occurring at the site of infection, or a catabolic process after CXCL10 has exerted its chemotactic potential.

## Materials and Methods

### Study Participants

For analysis of CXCL10 levels and DPP4 activity in plasma, we included 20 patients with microbiologically confirmed pulmonary TB (positive culture for *M. tuberculosis* from sputa) and 10 patients with pneumonia (either bacterial or viral) at the National Institute for Infectious Diseases, “Lazzaro Spallanzani” (INMI) in Rome, Italy. Patients were enrolled within 7 days of starting the specific treatment. Moreover 10 healthy controls were included from the CoSimmGEn cohort of the Investigation Clinique et Accès aux Resources Biologiques (ICAReB) platform (Centre de Recherche Translationnelle, Institut Pasteur in Paris, France). DPP4 enzyme activity was determined in 98 healthy controls included at Clinical Research Centre, Copenhagen University Hospital, Denmark. The baseline characteristics of these persons were previously described in the context of a TB diagnostic study (28).

For histopathology analysis, we collected formalin fixed, paraffin-embedded autopsy lung tissue samples from 25 subjects. These comprised: 9 patients with pulmonary TB (histologically, histochemically, or PCR-proven), 10 with pneumonia (viral or bacterial pneumonia), and 6 who died from other causes such as cardiac or renal failure. Autopsy lung tissue samples were fixed in neutral buffered-formalin immediately for histological and immunohistochemical analysis. For molecular studies, DNA was selectively extracted (QIAamp Tissue Kit, Qiagen GmbH, Hilden, Germany) from paraffin sections and PCR detection of *M. tuberculosis* DNA was performed.

For flow cytometry, patients with a clinical suspicion of TB undergoing bronchoalveolar lavage (BAL) as part of the diagnostic procedure for TB were considered for enrollment. According to the INMI institutional guidelines (http://www.inmi.it/protocolli), BAL is recommended if smear negative results were obtained from three sputa or two induced-sputa and no alternative diagnosis was set. TB was defined as “microbiologically confirmed,” if the culture from BAL was positive for *M. tuberculosis* and “clinical” if the BAL resulted negative to the *M. tuberculosis* culture but clinical, pathological, radiological findings consistent with TB were shown, and cure after specific treatment was demonstrated. Patients with pneumonia had a final diagnosis based on microbiological tests, clinical and radiological signs, and successful treatment with therapy different from TB-specific drugs.

### Ethics Statement

The project was approved by the Danish National Ethical Committee (H-3-2012-008); CoSimmGEn, Pasteur, France; and by the INMI Ethical Committee (parere no. 29/2014) Rome, Italy. All subjects provided written informed consent.

### Quantification of CXCL10 and Measurement of DPP4 Activity

Concentrations of all forms of CXCL10 (CXCL10^total^), agonist CXCL10 (CXCL10^long^), and antagonist CXCL10 (CXCL10^short^) were measured in Li-Hep and in protease-inhibited plasma using in-house Simoa ultrasensitive digital ELISA (Quanterix) (29). DPP4 activity was measured in a colorimetric assay in plasma, in brief, 150 µl of a 1 nM solution of the colorless DPP4 substrate H-Gly-Pro p-Nitroanilide (Sigma-Aldrich Co., St. Louis, MO, USA) was incubated for 2 h at 37°C with 5 µL EDTA plasma, and the generation of the yellow colored product p-Nitroanilide (p-Na) was detected at 405 with 630 nm as reference every 10 min. Measurements were compared to a set of samples with known p-Na concentration. DPP4-enzyme activity was calculated from the following equation: [((*Y* − *X*) × 0.155 nmol/well)/ΔT] × (1,000/5 μl), where *X* = *p*-Na concentration at *T*_X_ min; *Y* = *p*-Na concentration at *T*_Y_ min; ΔT = *T*_Y_ – *T*_X_ min, with one unit defined as the turnover of 1 µmol/min.

### Immunohistochemistry

Tissue samples were routinely fixed with neutral buffered 4% formalin (Bio Optica, Milan, Italy) for at least 48 h and routinely processed to paraffin blocks. Four micrometer sections were cut, mounted on coated slides, and dried overnight at 37°C. Sections were stained with hematoxylin and eosin. Immunostaining was performed on Benchmark XT system (Ventana, Tucson, AZ, USA). The following antibodies were used: CD3 rabbit monoclonal antibody (mAb) (2GV6, Roche, Ventana, Tucson, AZ, USA); CD4 rabbit mAb (SP35, Roche), CD8 rabbit mAb (SP57, Roche), CD14 rabbit Ab (EPR3653, Roche); CXCL10 rat mAb, clone IR2 (provided by Morten Ruhwald), CD26/DPP4 rabbit mAb (ab28340, Abcam); CXCR3/CD183 mouse mAb (BD Pharmigen). Positive reactions were visualized with peroxidase polymer labeled secondary antibody (Ultravision LP Value Detection System, Thermo Fisher Scientific, Waltham, MA, USA) and Value HRP polymer (Thermo Fisher Scientific). No double stainings were performed. The sections were evaluated by light microscopic examination and cellular localization and intensity of immunostaining in each section were assessed by two pathologist observers (Andrea Baiocchini and Franca del Nonno) using a 40× objective corresponding to a tissue area of 30 mm^2^. Results were evaluated considering the overall proportion of positive cells. Immunohistochemical scores were assigned taking into consideration the proportion of positive cells (scored on a scale of 0–4): score 0, no staining; score 1, from “0 to 10%” positive cells; score 2, from “11 to 30%” positive cells; score 3, from “31 to 70%” positive cells; score 4, if “>70%” positive cells.

### Confocal Microscopy

Deparaffinized and rehydrated sections were immersed in 1 mM EDTA solution, pH 8.0, and microwaved for antigen retrieval. Samples were briefly rinsed in phosphate-buffered saline (PBS) solution, permeabilized with 0.2% Tween in PBS, and blocked with 1% bovine serum albumin and 10% normal serum in PBS. Primary antibodies: mouse anti-CD4 mAb (NCL-L-CD4-1F6, Newcastle upon Tyne, UK), rabbit anti-CD14 (clone EPR3653, Roche), rat anti-CXCL10/IP-10 mAb (clone IR2, provided by Morten Ruhwald), were incubated over night at 4°C. After washing, samples were incubated with Jackson donkey anti-rat Cy3 antibody and Alexa488, Alexa647 fluorochrome-coupled goat secondary antibodies directed against rabbit or mouse. Coverslips were mounted in SlowFade-Anti-Fade (Invitrogen, Life Technologies). Fluorescence was analyzed with a TCS SP2 confocal laser scanning microscope (Leica Microsystems, Wetzlar, Germany). Digital images obtained through a 63× objective (zoom factor 2×) were acquired with Leica Confocal Software.

### Blood and BAL Processing for FACS and Plasma Measurements

Heparinized peripheral blood was collected and processed within 4 h. Peripheral blood mononuclear cells (PBMCs) were isolated by Ficoll density gradient centrifugation. Cells from BAL (BALC) were obtained by filtering the fluid through a sterile 100 µm cell strainer (BD Falcon, Erembodegem, Belgium) and then washing them with PBS. Plasma was collected from standard EDTA blood collection tubes (BD Biosciences) and protease inhibited plasma for CXCL10 isoforms was collected in P800 tubes (BD Biosciences).

### FACS

For analysis of CXCR3^+^ and CD26^+^ expression PBMC and BALC were counted and fresh stained for 15 min at 4°C with the live dead stain Aqua dye (Invitrogen Life Technology, Monza, Italy) according to the manufacturer’s protocol and then fixed in 2% paraformaldehyde. Then, the cells were resuspended in the PBS-2%-fetal calf serum 0.5% buffer and stained with mAbs for the following surface markers: anti-CD3 phycoerythrin (PE)-Cy7-conjugated (BioLegend, San Diego, CA, USA); anti-CD4 allophycocyanin eFluor-conjugated (eBioscience, San Diego, CA, USA); anti-CD8 Pacific Blue (BD Bioscience, San Jose, USA); anti-CXCR3 Alexa Fluor 647 (BioLegend); anti-CD26 PE (BD Biosciences). At least 300,000 cellular events were acquired using a FACSCanto II flow cytometer (BD Biosciences). Analysis was performed with FlowJo software (Tree Star Inc., San Carlos, CA, USA). Gating strategies are provided in Figure S1 in Supplementary Material.

### Statistics

Groups were compared using non-parametric methods (Kruskal–Wallis test, Spearman), and linear regression using the least squares method, using GraphPad Prism 6.0 (graph pad software, CA, USA). Pair wise comparisons were performed using Mann–Whitney test with Bonferroni correction. A *p*-value < 0.05 or <0.016 were considered significant.

## Results

### Patients With Active TB Have High Plasma Levels of CXCL10, Predominately in Antagonist Form

**Figure 1:**
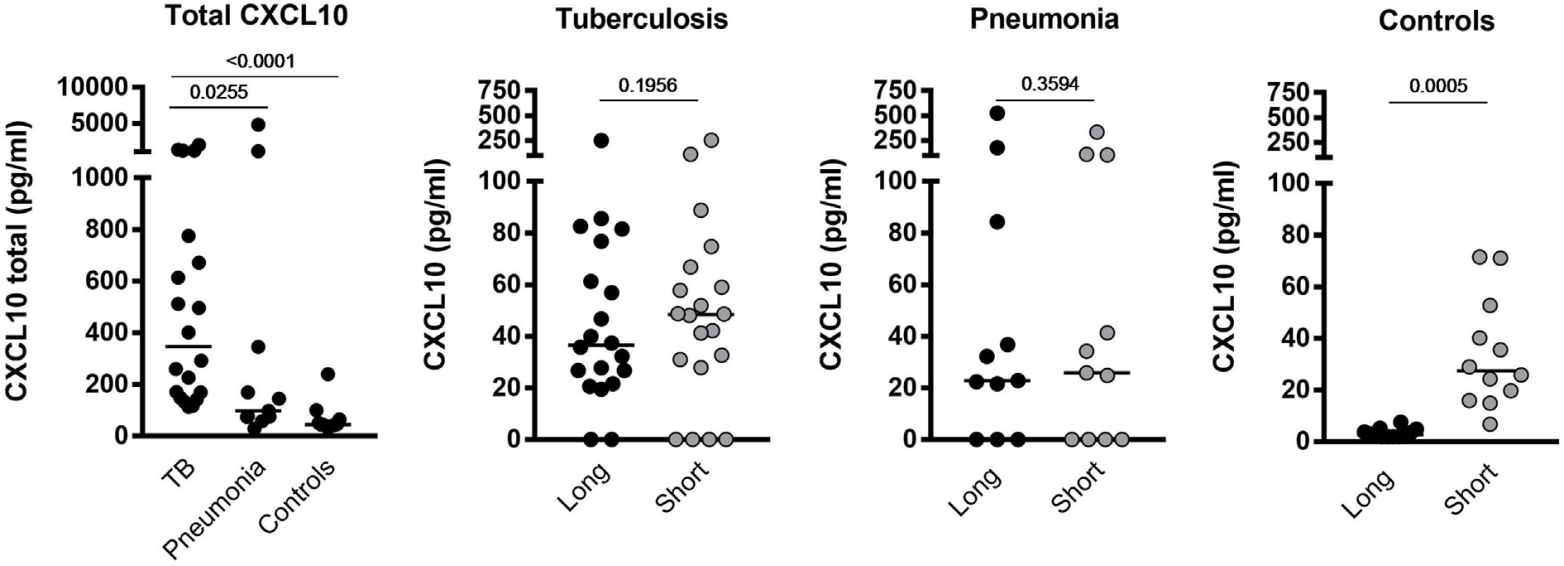
Plasma levels of CXCL10^total^, CXCL10^long^, and CXCL1010^short^ in samples from tuberculosis (TB) patients, pneumonia, and healthy controls.

We determined CXCL10 plasma levels in samples from 20 TB patients, 11 patients with bacterial pneumonia, and 12 healthy controls (Table 1). TB patients had significantly higher levels (*p* < 0.0255) of all forms of CXCL10 (CXCL10^total^) compared to that found in patients with pneumonia and controls (Figure 1). Using the Simoa assay, we differentiated between full length CXCL10 (CXCL10^long^) and the DPP4 truncated antagonist form (CXCL10^short^) in the samples from the three cohorts (Figure 1). TB patients had significantly higher level of CXCL10^long^ compared to controls (*p* < 0.0001), but there were no other significant differences in either CXCL10^short^ and CXCL10^long^ within the groups and between groups.

**Table 1:** Baseline information of study participants.

	Active TB	Pneumonia	Controls
*N*	20 (48.8)	11 (26.8)	10 (24.4)
Age median (IQR)	37 (31–47)	50 (32–65)	50 (31–61)
**Sex *N* (%)**			
Female	4 (20.0)	4 (36.4)	4 (40.0)
**Origin *N* (%)**			
West Europe	3 (15.0)	10 (90.9)	7 (70)
East Europe	13 (65.0)	1 (9.1)	1 (10.0)
Asia	2 (10.0)	0 (0)	0 (0)
Africa	1 (5.0)	0 (0)	1 (10.0)
America	1 (5.0)	0 (0)	1 (10.0)
**BCG *N* (%)**			
Vaccinated	17 (85.0)	3 (27.3)	1 (10.0)
**QFT *N* (%)**			
Positive	13 (65.0)	0 (0)	4 (40.0)
Negative	3 (15.0)	6 (54.5)	5 (50.0)
Indeterminate	1 (5.0)	0 (0)	1 (10.0)
Not done	3 (15.0)	5 (45.5)	0 (0)

*N, number; TB, tuberculosis; IQR, interquartile range; BCG, Bacillus Calmette–Guérin; QFT, QuantiFERON Gold In Tube*.

**Figure 2:**
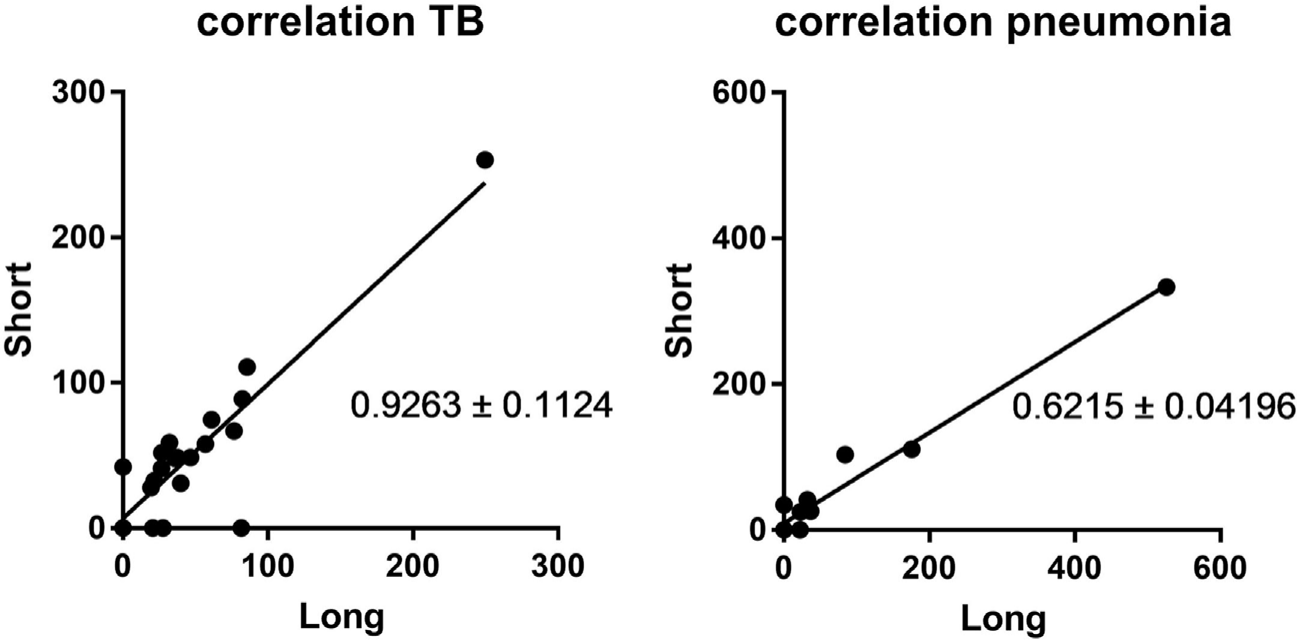
Correlation between CXCL10 short and long. Left panel: samples from the active tuberculosis patients; right panel: samples from pneumonia patients.

CXCL10^short^ and CXCL10^long^ levels were highly correlated in the two patient groups (*r*^2^ = 0.81 and 0.68, *p* < 0.0001, Spearman), of note, the correlation revealed a significant difference in the regression slopes of TB patients (0.92 SE ± 0.11) compared to pneumonia patients (0.62 SE ± 0.04, *p* < 0.0001), suggesting that CXCL10 was more readily truncated to the short form in TB patients (Figure 2).

### Plasma DPP4 Activity Is Lower in TB Patients Compared to Controls

**Figure 3:**
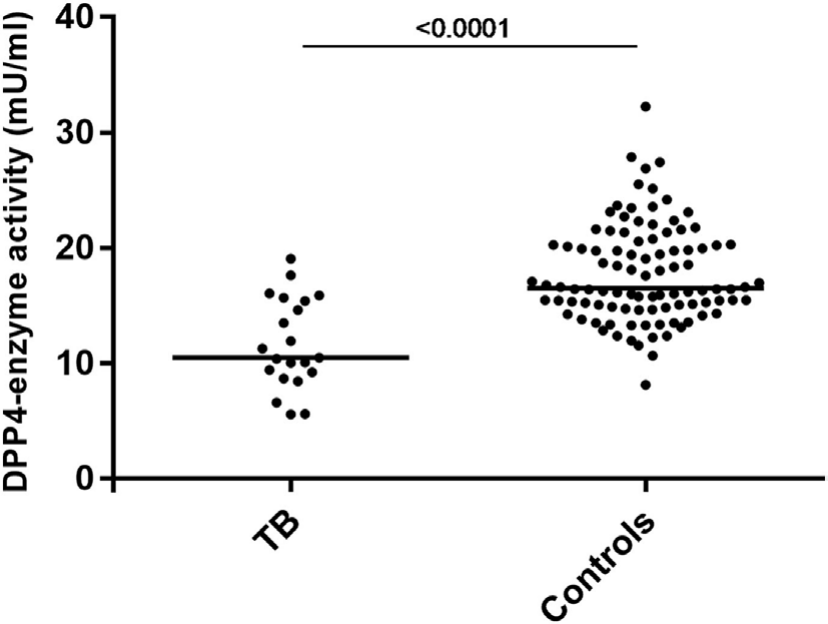
Decreased plasma DPP4 enzyme activity in tuberculosis (TB) patients. DPP4 enzyme activity in plasma determined using colometric assay in 21 patients with active TB and 98 healthy controls.

To determine if the high levels of CXCL10^short^ in TB patients were due to an increased activity of soluble DPP4 in plasma, we measured the enzymatic activity of DPP4 in LiHep-plasma samples from 21 TB patients and 98 healthy controls. Interestingly, the DPP4 activity was lower in TB patients [median 10.5 mU/ml, interquartile range (IQR) 9.0–15.5], compared to healthy controls [16.5 (15.0–21.0), *p* < 0.0001], suggesting that CXCL10 N-terminal truncation in TB patients may occur before CXCL10 enters the bloodstream (Figure 3).

### Analysis of CXCL10 Expression and Characterization of Inflammatory Cell Types in Lung, at Site of TB Infection, in BAL and in PBMC

**Figure 4:**
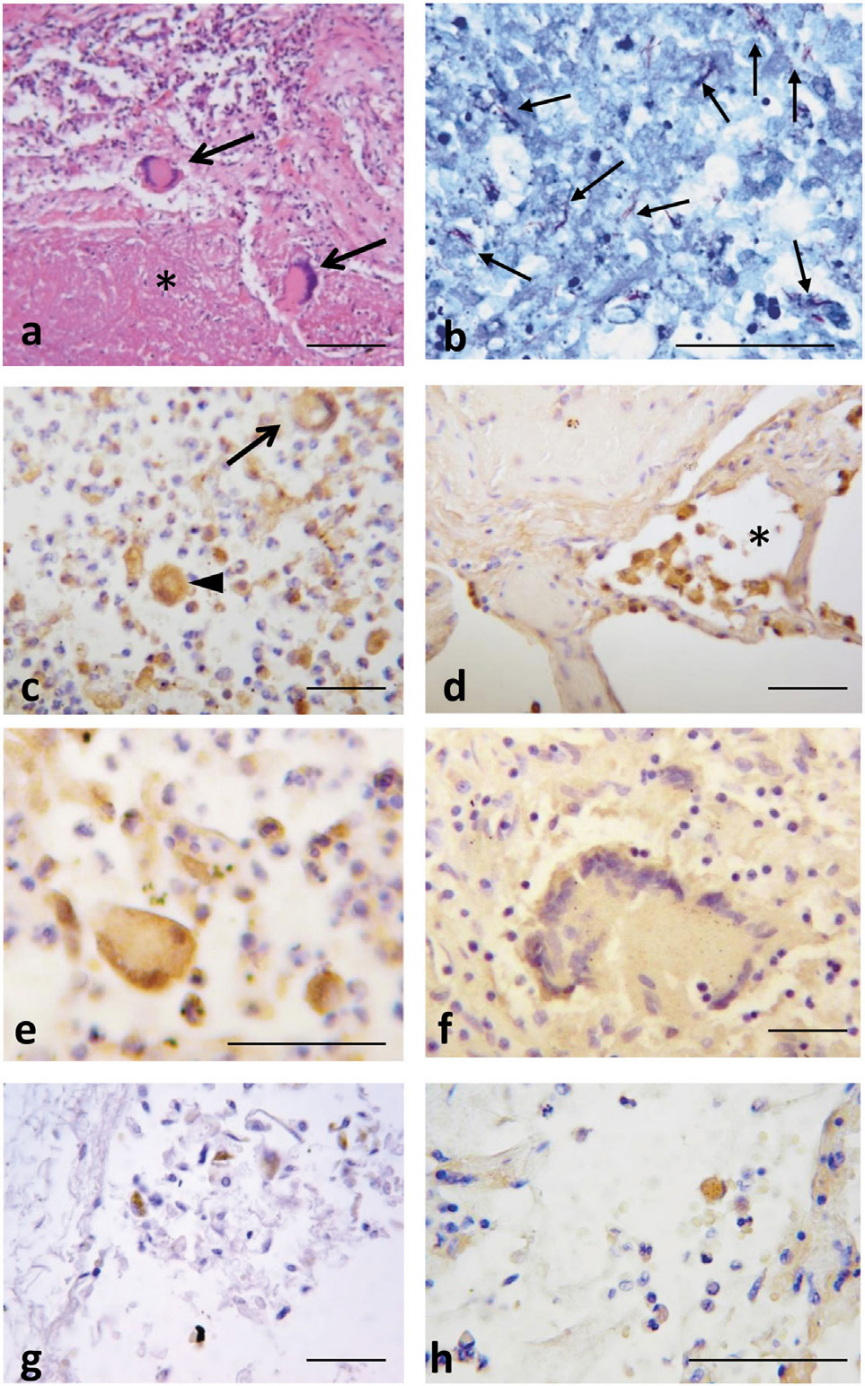
Lung tuberculosis (TB) histopathology and CXCL10 staining in post-mortem samples from patients with active TB, pneumonia, or causes different from active TB. **(A)** Representative histological image of a tubercle rich with caseous necrosis (*), and Langhans’ cells (arrows). **(B)** Numerous acid fast bacilli (in red, arrows) demonstrate the presence of Mtb after Ziehl–Neelsen acid-fast stain. **(C)** CXCL10 expression in hystiocytes (arrow head), multinucleated giant cells (arrow), and other inflammatory cells; **(D)** in the contiguous lung parenchyma, CXCL10 staining was detected in macrophages inside the alveolar space (*). **(E,F)** Multinucleated giant cells display intense or weak CXCL10 positivity. In control samples **(G)** and in pneumonia **(H)**, CXCL10 expression is found in macrophages. Scale bars: **(A)** = 100 µm; **(B–H)** = 50 μm (based on the single length of the indicated bar).

Immunohistochemical assays were performed to evaluate CXCL10 expression in lung specimens taken post-mortem from patients with active TB (mycobacterial DNA demonstrated by PCR, data not shown), pneumonia and patients with diffuse alveolar damage related to cardiac failure (Table 2). The classic tuberculoid granuloma formed by a large zone of caseous necrosis bordered by palisading histiocytes and multinucleated giant cells (Langhans’ giant cells) was observed in lungs from TB patients (Figure 4A). Mycobacteria were demonstrated using the Ziehl–Neelsen acid-fast stain (Figure 4B). In these samples, CXCL10 expression was seen in histiocytes and giant cells in granulomas (Figure 4C) and in macrophages inside alveolar spaces in the contiguous lung parenchyma (Figure 4D). Multinucleated giant cells showed both strong and weak staining intensity for CXCL10 (Figures 4E,F). In controls, CXCL10-positive staining was found in macrophages in alveolar spaces (Figure 4G), while in pneumonia samples, CXCL10-positive histiocytes were found in inflammatory exudates (Figure 4H). The quantitative assessment of CXCL10 showed high labeling score (score 4 in majority of cases) in TB with respect to controls and pneumonia cases (Figure S2 in Supplementary Material).

**Table 2:** Demographical and clinical characteristics of the patients whose autopsies samples were evaluated by immunohistochemistry.

	Active TB	Pneumonia	Controls
*N*	9	10	6
Age median (IQR)	41 (30–57)	50 (40–59)	56 (43–77)
**Sex *N* (%)**			
Female	4 (44.4)	3 (30.0)	3 (50.0)
**Origin *N* (%)**			
West Europe	3 (33.3)	7 (70.0)	3 (50.0)
East Europe	3 (33.3)	3 (30.0)	2 (33.3)
Asia	1 (11.1)	–	1 (16.7)
Africa	1 (11.1)	–	–
America	1 (11.1)		
**BCG *N* (%)**			
Vaccinated	6 (66.7)	3 (30.0)	3 (50.0)
**Diagnosis *N* (%)**			
Endocarditis	–	–	3 (50.0)
Sepsis	–	–	1 (16.7)
Renal failure	–	–	1 (16.7)
Pulmonary edema	–	–	1 (16.7)

*N, number; TB, tuberculosis; BCG, bacillus Calmette-Guérin; IQR, interquartile range; QFT, QuantiFERON TB Gold In tube; TST, tuberculin skin test*.

**Figure 5:**
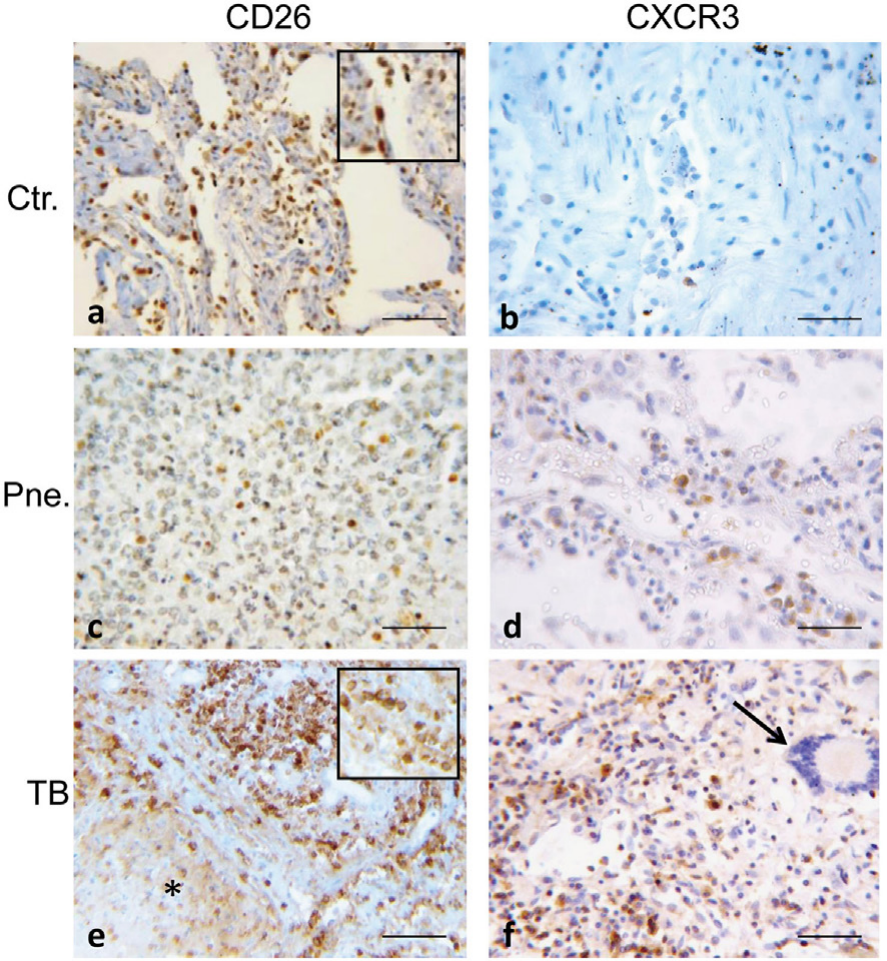
Representative images of normal control lung (Ctr), of pneumonia (Pne) and necrotic and inflammatory zones of tuberculosis (TB) are immunostained for CD26 (DPP4) and CXCR3 (CD183). **(A)** In control lung, many CD26-positive cells are visible, consisting not only in lymphocytes but also in mesenchymal/endothelial cells lining alveolar space (magnified image in the insert shows CD26-positive mesenchymal/fibroblast cell). **(C)** In pneumonia, rare positive cells corresponding morphologically to lymphocytes, are shown. **(E)** Strongly CD26-stained cells are clustered in inflammatory area surrounding necrotic lesion of TB lung; they appear morphologically consistent with lymphocytes (as seen in magnified image in the insert). **(F)** CXCR3 is strongly expressed in lymphocytes in the inflammatory region of tuberculous lesion; on the right a multinucleated giant cells (arrow) not labeled **(B,D)**. Rare CXCR3 expressing cells are present both in pneumonia and in controls. Scale bars: **(A–F)** = 50 μm.

The expression of CD26/DPP4 and of CXCR3/CD183 were further analyzed on the same samples (Figure 5). In TB cases numerous strongly positive CD26^+^ cells were clustered in inflammatory cellular reactions due to necrotizing granuloma (Figure 5E), while in pneumonia, spread positive cells were observed in exudates (Figure 5C). In controls CD26 positivity was not limited to lymphocytes but it was also found in the fibroblast population in the alveolar septa (Figure 5A). The results of CXCR3 immunostaining showed rare CXCR3 expressing cells, both in pneumonia and in controls (Figures 5B,D). Intense positivity for CXCR3 was observed in lymphocytes localized in the inflammatory region of tuberculous lesions, whereas multinucleated giant cells did not express CXCR3 (Figure 5F). The quantitative evaluation of CD26 and CXCR3 showed a range of scores between 2 and 4 in TB lung, with the majority of positivity within score 3; both in pneumonia and controls the majority of cases displayed a CD26 score between 1 and 2, and a CXCR3 score between 0 and 2 (Figure S2 in Supplementary Material).

**Figure 6:**
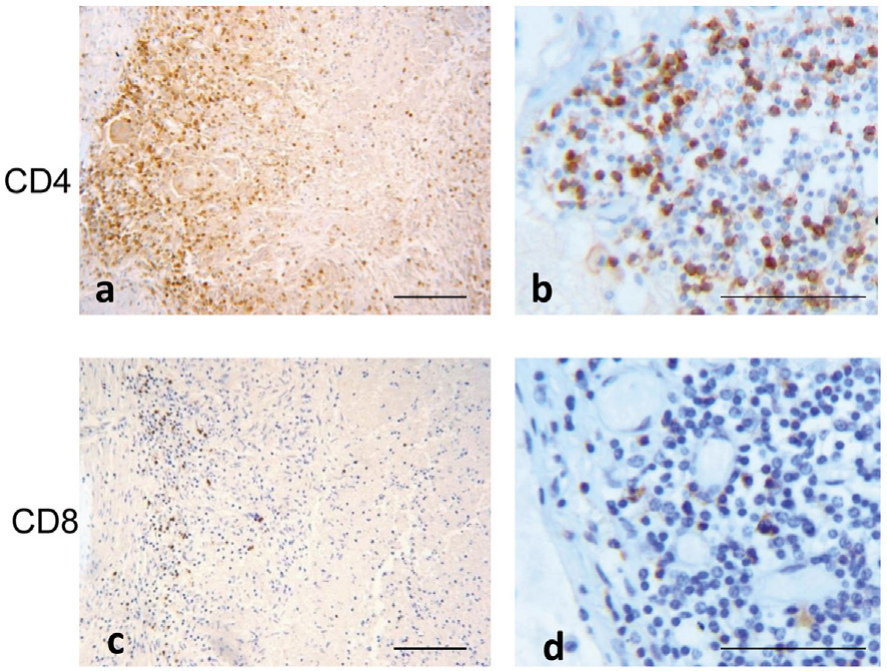
T cell phenotype distribution in tuberculous lesion. Immuno-staining with anti-CD4 **(A,B)**: low and high magnification and anti-CD8 **(C,D)**: low and high magnification show a prevalence of CD4 T lymphocytes. Scale bars: **(A,C)** = 100 μm; **(**B,D) = 50 μm.

**Figure 7:**
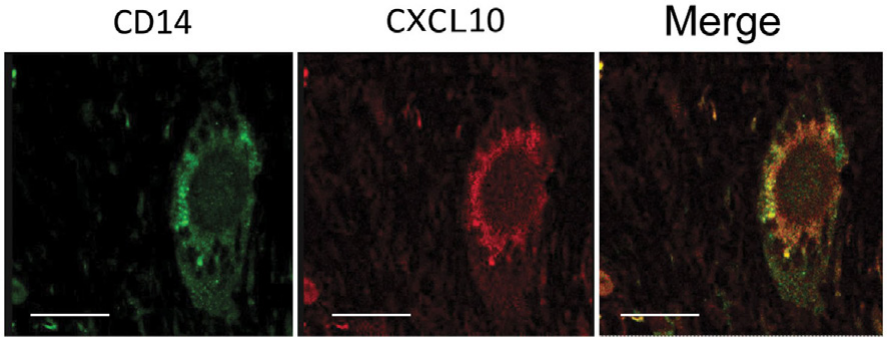
CXCL10 expression in multinucleated giant cells. Confocal microscopy analysis of tuberculosis lung sample immunolabeled for CD14 (green) and CXCL10 (red) revealed the presence of CXCL10 in Langhans’ cells, as visible by the co-localization of the two markers in the merged image (merge). Scale bar: 16 µm.

**Figure 8:**
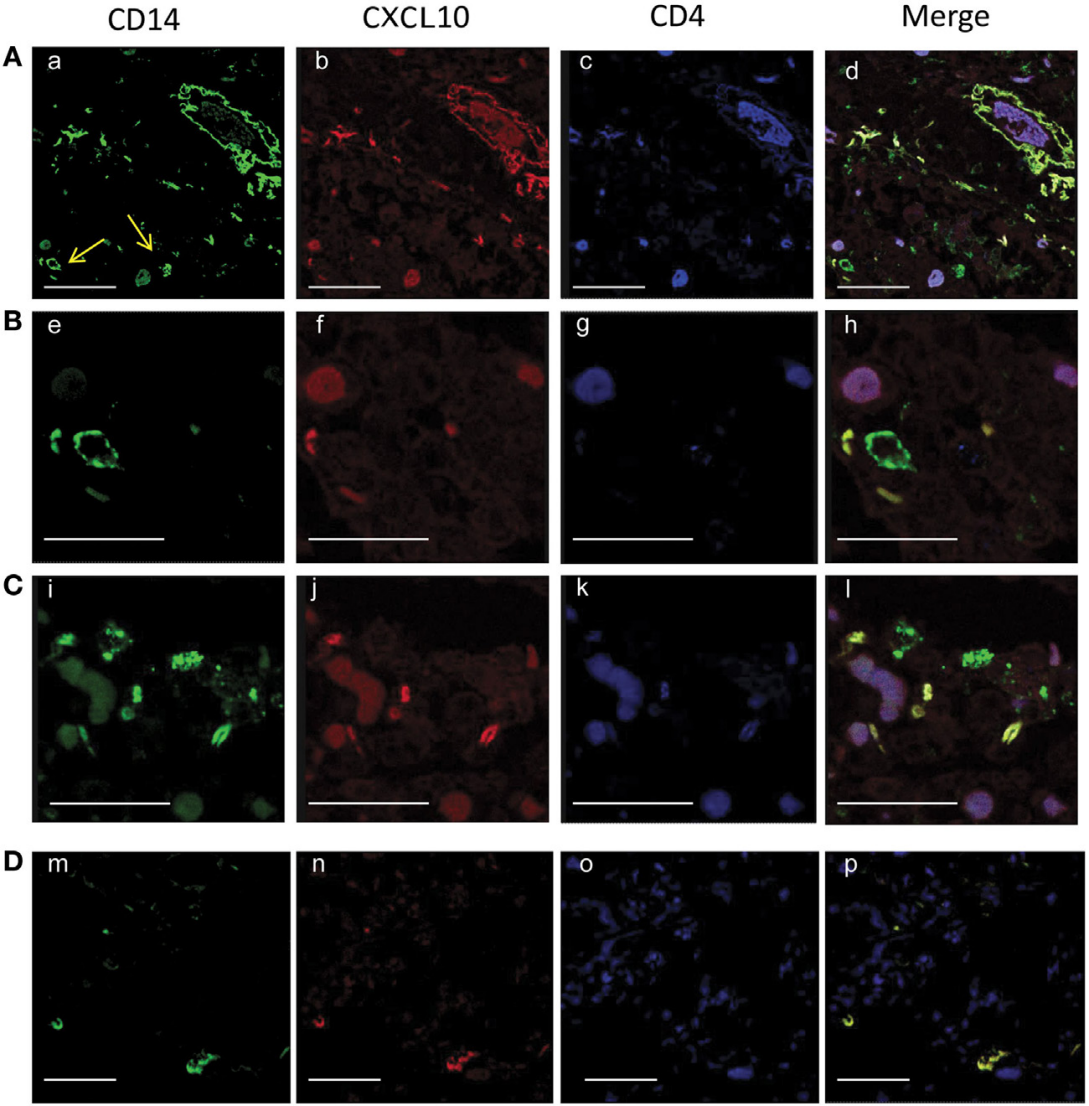
Confocal microscopy analysis of CXCL10 expression in tuberculosis (TB) [lines, **(A–C)**] and control [line, **(D)**] samples. Lung sections were stained with anti-CD14 (a, e, i, m, green), with anti- CXCL10 (b, f, j, n, red), and with anti-CD4 (e, g, k, o, blue). In TB samples, the merged images of the triple-fluorescence signals (d, h, l, *m*erge) highlights the expression of CXCL10 in CD4^+^ T lymphocytes (purple) presents in the inflammatory area and in macrophages (yellow); some CD14^+^ positive cells do not express CXCL10 (green). In controls, merged image (p) shows the CXCL10 expression only in CD14^+^ positive cells (yellow). Scale bars: (a, d) (m−p) = 25 µm; (e−h) = 8 µm.

The majority of recruited lymphocytes were CD4 T lymphocytes, as demonstrated by the positive immunohistochemical analysis (Figures 6A,B) with fewer sparsely distributed CD8^+^ cells (Figures 6C,D). Immunofluorescence analysis performed by confocal microscopy showed that multinucleated giant cells were associated to CXCL10 production, as demonstrated by the co-localization with CD14 (Figure 7). In the other inflammatory cells, present around the necrotic region, CXCL10 co-localized also with CD4^+^ T lymphocytes (Figure 8, line **B**); regarding macrophages, some of the CD14^+^ cells scored CXCL10 positive (Figure 8, line **C**), while other macrophages were negative (Figure 8, lines **A–C**). In contrast, in control samples, CXCL10 was found co-localized only with CD14^+^ cells (Figure 8, line **D**).

**Figure 9:**
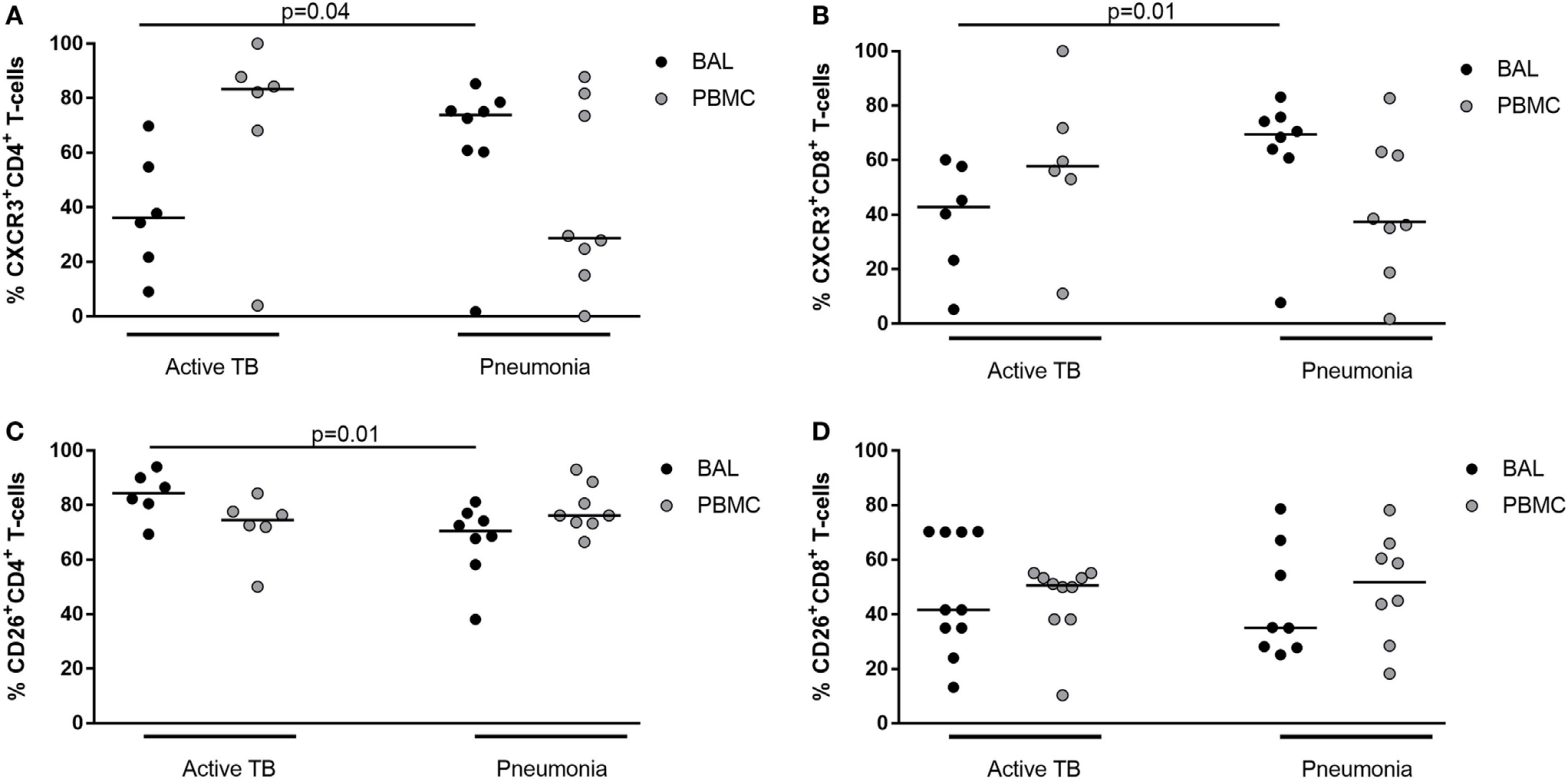
Frequency of CD4 and CD8^+^ CXCR3^+^ or CD26^+^ T-cells in bronchoalveolar lavage cells and peripheral blood mononuclear cells from patients with active tuberculosis (TB) and pneumonia.

Moreover, the expression of CXCR3 and CD26 was evaluated in BALCs and PBMCs by flow cytometry in six patients with active TB and eight patients with pneumonia (Table 3; Figure 9). The results showed that in TB patients, CXCR3 expression in CD4^+^ and CD8^+^ cell subsets was lower in the lung compared to the periphery, whereas in pneumonia, the CXCR3 expression in CD4^+^ and CD8^+^ cell subsets was lower in the periphery compared to the lung (Figures 9A,B). Moreover, patients with active TB showed a significantly lower expression of CXCR3 in the lung compared to patients with pneumonia in both CD4^+^ and CD8^+^ cell subsets (Figures 9A,B). Conversely, CD26 expression in the lung of active TB patients was significantly higher than that found in pneumonia, at least in the CD4^+^ cell subset (Figures 9C,D).

**Table 3:** Demographical and clinical characteristics of patients evaluated by flow cytometry.

	Active TB	Pneumonia
*N*	6	8
Age median (IQR)	46 (34–60)	51 (44–56)
**Sex *N* (%)**		
Female	5 (83.3)	7 (87.5)
**Origin *N* (%)**		
West Europe	3 (50.0)	7 (87.5)
East Europe	2 (33.3)	–
Asia	1 (16.7)	–
Africa	–	1 (12.5)
**BCG *N* (%)**		
Vaccinated	3 (50.0)	1 (12.5)
**TST *N* (%)**		
Positive	3 (50.0)	3 (37.5)
Negative	2 (33.3)	3 (37.5)
Not done	1 (16.7)	2 (25.0)
**QFT *N* (%)**		
Positive	3 (50.0)	2 (25.0)
Negative	1 (16.7)	4 (50.0)
Indeterminate	1 (16.7)	–
Not done	1 (16.7)	2 (25.0)
**TB diagnosis *N* (%)**		
Microbiological	2 (33.3)	–
Clinical	4 (66.7)	–

*N, number; TB, tuberculosis; BCG, bacillus Calmette-Guérin; IQR, interquartile range; QFT, QuantiFERON TB Gold In tube; TST, tuberculin skin test*.

## Discussion

High CXCL10 blood levels are a hallmark of many chronic infections and assumed to reflect ongoing recruitment of antigen-specific cells to the site of inflammation through CXCR3 chemoattraction (20). Casrouge and colleagues showed that CXCL10 in plasma from patients with chronic HCV infection predominantly exists in an antagonist form, hereby explaining the apparent paradox that the most potent chemokine directing HCV virus specific Th1 cells to the infected liver is also a strong negative predictor for the failure to respond to IFN-based treatment (22). Prompted by these results, we investigated whether a similar phenomenon may be involved in TB pathogenesis.

In line with several other publications, we showed that CXCL10 is increased in the blood of patients with active TB and pneumonia, as compared to healthy volunteers (14, 16, 18, 28). We further dissected the relative contribution of agonist and antagonist forms of CXCL10 and found that the antagonist form was present in high levels in plasma of active TB patients. We identified a source of CXCL10 production at the site of infection in the lung, where *M. tuberculosis*-infected alveolar macrophages secrete high levels of CXCL10. To investigate the site of CXCL10 truncation, we found low DPP4 enzymatic activity in plasma of active TB patients, but high levels of CD26/DPP4 expression in the lung tissue presenting the hallmark pattern of TB lesions, with a lymphocyte collar surrounding necrotic nodule. At the site of the pathologic response to the disease, relative expression of CXCR3 was higher compared to patients with pneumonia of different etiology. Around parenchymal lesions, multinucleated giant cells co-expressed CXCL10 as revealed by confocal microscopy analysis. Immunofluorescence assays showed that both CD4^+^ positive T cells and macrophages co-localize with CXCL10 in the site of granulomatous inflammation. Taken together, the findings suggest a novel—potentially central—regulatory role of DPP4 in TB, through the inhibition of Th1 T cell homing to the TB-infected lung.

Our data bridges previous observations of high levels of CXCL10 and CD26 in TB pleural effusions (30) and of CXCL10 expression in lungs of non-human primates experimentally infected with *M. tuberculosis* (13). These findings may potentially be extended to other granulomatous infections, e.g., tuberculoid leprosy also characterized by high levels of CXCL10 and CD26 expression at the site of infection (31, 32). We observed a striking lower plasma activity of soluble DPP4 in TB patients compared to healthy individuals further supporting the concept that CXCL10 truncation is an early event occurring at the site of infection. A similar phenomenon has been observed in some autoimmune, inflammatory, and infectious diseases, as well as in several cancers; however, no link between plasma DPP4 activity and chemokine antagonism has been shown and the individual role of membrane-bound and soluble DPP4 remains poorly understood (25, 33, 34).

### New Opportunity for HDT?

Our finding that DPP4 could act as a negative regulator of CXCR3-mediated Th1 cell homing to the site of *M. tuberculosis* infection, opens a potential new opportunity for host-directed therapy (35). Selective DPP4 inhibitors are marketed for the treatment of type 2 diabetes, and—as shown by us and others—this class of drugs can abrogate the truncation of CXCL10 by DPP4 *in vitro* and *in vivo* (22, 24). These findings were recently extended in a clinical trial demonstrating the rapid *in vivo* abrogation of CXCL10 truncation in both healthy controls and HCV patients treated with daily 100 mg Sitagliptin (Januvia^®^)—hereby, establishing a proof-of-concept for intervention trials (23). DPP4 inhibitors are already being considered as repurposed immunotherapeutic drugs in oncology with promising results. Proof-of-concept in a mouse model showed that the Sitagliptin-mediated DPP4 inhibition enhanced the tumor rejection by preserving the agonist form of CXCL10 and increasing the trafficking into the tumor by the CXCR3 expressing lymphocytes (24).

### DPP4 a Regulatory Mechanism Dampening Th1 Overdrive?

Human Th1 cells specific for *M. tuberculosis* are largely contained in a CXCR3-expressing subset (12) and in animal models it has been shown that CXCR3-expressing T cells are essential for containment of the infection during natural infection and by the T cells induced in experimental vaccines (36–39). It is thus tempting to speculate on repurposing of DPP4 inhibitors for host-directed therapy of human TB to improve migration of the relevant cells to the infection site. This approach has potential as an adjunct to standard antibiotic treatment, where an increased number of *M. tuberculosis*-specific T cells could eliminate persistent bacilli thought to be in a metabolic state less susceptible to killing by drugs (40). The decreased regulation of Th1 cell homing to the lung of TB patients could backfire as an inflammatory overdrive potentially increase inflammation, tissue destruction, and risk of cavernous TB (37); however, the DPP4 class of drugs are considered safe (41) and meta-analysis of post marketing data have not suggested an increased risk (42).

This study also suggests a new avenue for TB disease monitoring. CXCL10 is already an established biomarker for TB, where high levels are associated with extent of infection and treatment efficacy (14, 18, 19, 28). Further refining this biomarker to directly quantify functional or non-functional forms could deliver more informative clinical information; however, such applications would require further studies.

### Limitations

Our study is descriptive, and the key claim that the abrogated CXCR3/CXCL10 axis is DPP4-mediated and occurring at the site of infection remains circumstantial without direct confirmation. Unfortunately, the specific Abs for short and long forms of CXCL10 are not functional in the histological assays; therefore, a likely next step would be confirmation in a small animal model. Unavailability of plasma samples collected in protease inhibited blood collection tubes from controls prevented a direct correlation of DPP4 activity and plasma levels of CXCL10 forms in the same samples. We compensated for this limitation by including a larger cohort of controls. Moreover, as the data strongly suggest that DPP4 truncation happens before CXCL10 enters the blood, we consider this missing link in paired samples of lesser importance. We observed high levels of tissue auto-fluorescence in the confocal microscopy analysis in Figure 8. This is a well know challenge caused by lung tissue features such as collagen, lipofuscin, elastin, and red blood cells (43), in this case, aggravated by the quality of the biopsies from human cadavers obtained several hours post-mortem and fixed with 10% neutral-buffered formalin embedded in paraffin (44). Due to these inherent limitations, immuno-fluorescence staining of this type of samples is not a routinely used method in clinical pathology (44); however, as these are very rare specimens not easily obtained for an exploratory project, we have included them in the project and highlighted the limitations.

## Conclusion

Taken together, our work provides the first evidence of post translational CXCL10 processing in human TB. The data suggest that in TB, CXCL10 is secreted by *M. tuberculosis*-infected macrophages, and undergoes rapid inactivation by membrane bound DPP4 (CD26) expressed on co-localized CD4^+^ T cells. As only few of the abundant number of CD4^+^ T cells at the site of infection express CXCR3, it seems plausible that the CXCL10/CXCR3 chemotactic axis is under regulation by DPP4 in active TB. The regulatory mechanism suggests a double-edged sword beneficial for the host by preventing inflammatory overdrive as well as for the bacteria by limiting T cell-mediated elimination of the intracellular infection. Our findings further suggest a potential novel avenue for host-directed therapy as DPP4 inhibitors used in the treatment of diabetes could be refurbished for patients with TB and MDR-TB. This warrants further exploration.

## Ethics Statement

The project was approved by the Danish National Ethical Committee (H-3-2012-008); CoSimmGEn, Pasteur, France; and by the INMI Ethical Committee (parere no. 29/2014) Rome, Italy. All subjects provided written informed consent.

## Author Contributions

TB developed in-house assays for DPP4 measurement, conducted FACS analysis on PBMCs, analyzed and interpreted data, participated in the inclusion of controls and co-wrote the first draft of the manuscript; DG included patients, coordinated sample handling, contributed to the design of the study, coordinated the work done by cytometry and on autopsy samples; participated in the interpretation of data, contributed to the writing of the manuscript; FN, LF, and AB performed the histological analysis, immunohistochemistry, confocal microscopy analysis, and data interpretation; AC participated in the development and optimization of assays and interpretation of data; LP conducted FACS analysis on PBMCs and BALC, analyzed and interpreted data, revised the manuscript; TC conducted FACS analysis on PBMCs and BALC, VV processed blood samples, JE Supervised the development of in-house assays and participated in the interpretation of data; MA and DD developed reagents for specific detection of CXCL10 forms, supervised assay development, and participated in the interpretation of data and preparation of the manuscript; VB developed and optimized Simoa CXCL10 assays and analyzed patient samples using SIMOA; MR coordinated the project, analyzed and interpreted data, and co-wrote the first draft of the manuscript. All authors approved the final version of the manuscript.

## Conflict of Interest Statement

MA is an employee of Genentech Inc., a member of the Roche group. The remaining authors declare that the research was conducted in the absence of any commercial or financial relationships that could be construed as a potential conflict of interest.

## Supplementary Material

Figure S1 Gating strategy of Broncho alveolar lavage, left (red box) cells and peripheral blood mononuclear cells, right (black box). Lymphocytes, singlets were gated based on FSC and SSC, followed by a CD3-positive gate (top panel). CD3^+^ cells were subdivided into CD4 or CD8 positive cells, and stained for CXCR3 high (bottom center), and CD26 high (bottom right).

Figure S2 Quantitative assessment of immunohistochemistry. Scores were assigned taking into consideration the proportion of positive cells on a scale of 0–4: score 0, no staining; score 1, from “0 to 10%” positive cells; score 2, from “11 to 30%” positive cells; score 3, from “31 to 70%” positive cells; score 4, if “>70%” positive cells.
