# Supplementary figures and images for: Interplay of DDP4 and IP-10 as a Potential Mechanism for Cell Recruitment to Tuberculosis Lesions
Source: Front Immunol. 2018 Jul 5;9:1456. doi: 10.3389/fimmu.2018.01456 (PMC6041415; doi:10.3389/fimmu.2018.01456)

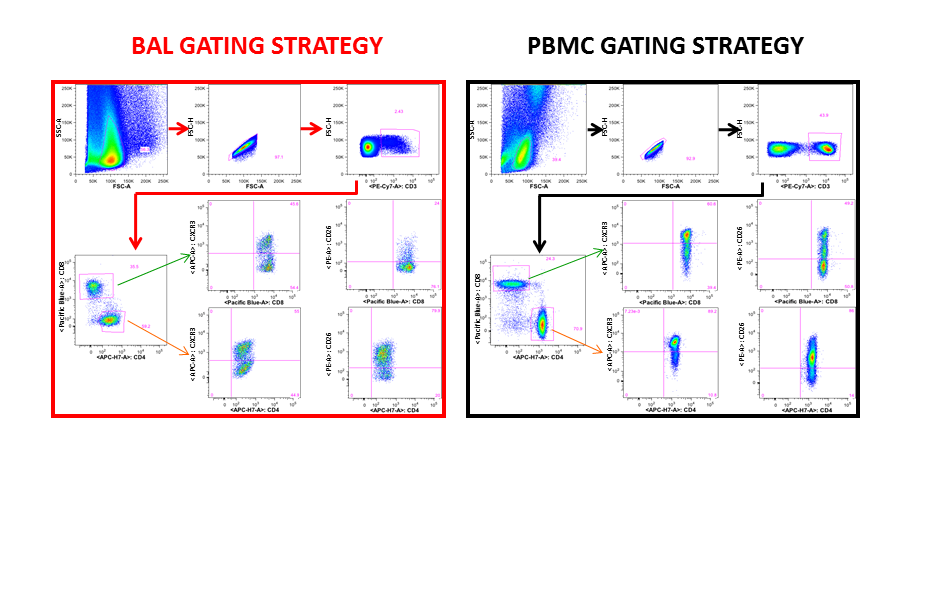

Supplement: Figure S1 — Gating strategy of Broncho alveolar lavage, left (red box) cells and peripheral blood mononuclear cells, right (black box). Lymphocytes, singlets were gated based on FSC and SSC, followed by a CD3-positive gate (top panel). CD3+ cells were subdivided into CD4 or CD8 positive cells, and stained for CXCR3 high (bottom center), and CD26 high (bottom right). [file image_1.tif]

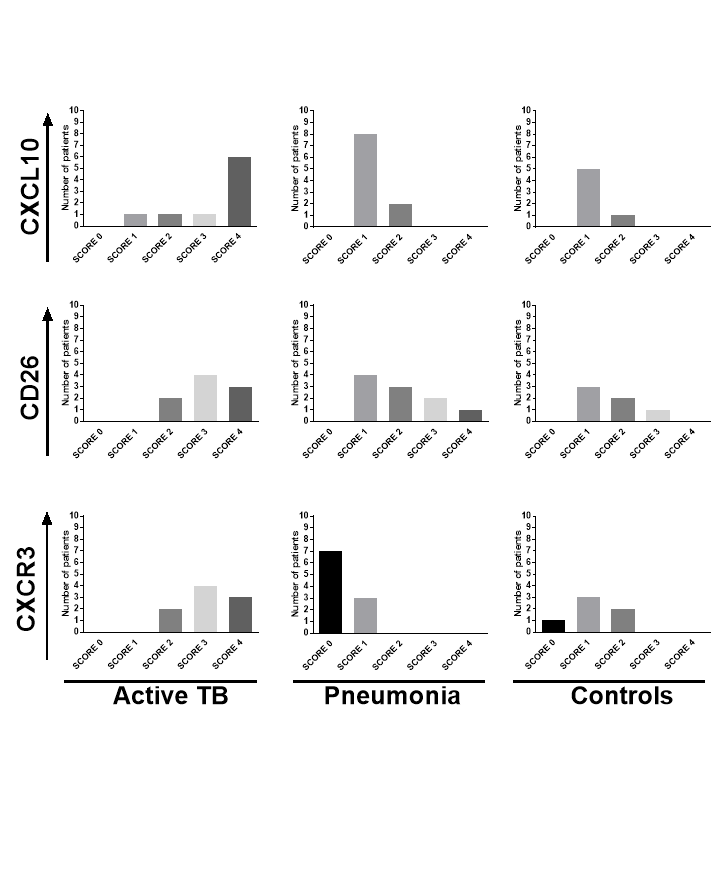

Supplement: Figure S2 — Quantitative assessment of immunohistochemistry. Scores were assigned taking into consideration the proportion of positive cells on a scale of 0–4: score 0, no staining; score 1, from “0 to 10%” positive cells; score 2, from “11 to 30%” positive cells; score 3, from “31 to 70%” positive cells; score 4, if “>70%” positive cells. [file image_2.tif]
